# A Multilevel Analysis of Job Characteristics, Emotion Regulation, and Teacher Well-Being: A Job Demands-Resources Model

**DOI:** 10.3389/fpsyg.2018.02395

**Published:** 2018-11-29

**Authors:** Hongbiao Yin, Shenghua Huang, Lijie Lv

**Affiliations:** ^1^Faculty of Education, Chinese University of Hong Kong, Hong Kong, China; ^2^Faculty of Education, Northeast Normal University, Changchun, China

**Keywords:** emotional job demands, trust in colleagues, emotion regulation, teacher well-being, the job demands-resources model

## Abstract

This study integrated personal factors into the job demands-resources (JD-R) model to examine school- and individual-level predictors of teacher well-being. Survey data were gathered from 1,656 teachers from 54 schools. The results of hierarchical linear modeling indicated that the school-level emotional job demands of teaching and suppression at the individual level were positively related to teachers' anxiety and depression whereas school-level trust in colleagues and individual-level reappraisal were positively associated with enthusiasm and contentment. Positive relationship between emotional job demands and suppression was also found. These findings support the claim that reappraisal should be considered a personal resource and suppression a personal demand.

## Introduction

The job demands-resources (JD-R) model developed by Bakker and his colleagues (Demerouti et al., 2001; Bakker and Demerouti, 2007, 2014, 2017; Schaufeli and Taris, 2014) is a broadly defined and widely used conceptual framework for understanding individuals' well-being and performance in the workplace. In this framework, all work environments and job characteristics fall into two general categories: job demands and job resources. The balance or imbalance between job demands and job resources is critical for predicting an individual's engagement or burnout, which in turn relates to work performance and health problems (Schaufeli and Bakker, 2004). However, most studies using the JD-R model have been conducted in fields other than education. With a very few exceptions (e.g., Hakanen et al., 2006; Simbula, 2010; Yin et al., 2016), the JD-R model has not been used to examine teacher well-being and its predictors in school settings.

It is widely recognized nowadays that teaching is an emotional endeavor (Chang, 2009; Sutton and Harper, 2009; Yin and Lee, 2012). Teacher emotion and the classroom emotional climate play critical roles in learning and teaching (Hosotani and Imai-Matsumura, 2011; Reyes et al., 2012; Hagenauer et al., 2015). In recent years, researchers have paid increasing attention to teachers' emotion regulation in the classroom and its effects on teacher well-being and student learning (Sutton, 2004; Fried, 2011; Chang, 2013; Yin, 2016). According to Gross (1998, 2015), individuals use two general strategies to regulate their emotions: cognitive reappraisal, an antecedent-focused emotion regulation strategy that involves reappraising emotion-eliciting situations before the arousal of emotions, and expressive suppression, a response-focused regulation strategy that involves inhibiting emotional tendencies once the emotion has already been generated. In the context of schooling, teachers who engage in reappraisal strategies may cognitively alter their perceptions of emotion-eliciting situations, such as disturbing student behaviors, and focus their attention on the bright sides of classroom interactions. In contrast, when teachers adopt suppression strategies, they modify the external expressions of their feelings by feigning positive emotions while hiding negative emotions (Hosotani and Imai-Matsumura, 2011; Yin, 2016).

The research on teachers' emotion regulation has been increasing during the past decade (Tsouloupas et al., 2010; Keller et al., 2014). It has been reported that school leaders' use of reappraisal is a better predictor of teachers' job satisfaction and emotional exhaustion than their use of suppression (Kafetsios et al., 2012) and that teachers' use of reappraisal is more effective than suppression at enhancing positive emotions and reducing negative expressions in the classroom (Jiang et al., 2016). As for the effects of emotion regulation on teacher well-being, Chang (2009, 2013) suggested that the positive relationship between suppression and burnout was significant while the relationship between reappraisal and burnout was not.

Teacher well-being was assessed by Warr's (1990) affective well-being model, which has been repeatedly suggested as a valid and comprehensive approach to defining and assessing individuals' well-being in the workplace (de Jonge and Schaufeli, 1998; Mäkikangas et al., 2007). According to Warr (1990), affective well-being should be conceptualized along two dimensions: “pleasure” and “arousal.” His model thus consists of two axes of anxiety-contentment and depression-enthusiasm: anxiety reflects an unpleasant and activated psychological status, while contentment is a pleasant and deactivated state; depression reflects an unpleasant and deactivated psychological status, while enthusiasm is an activated and pleasant state.

The multilevel analysis is an unresolved issue in JD-R theory (Schaufeli and Taris, 2014; Bakker and Demerouti, 2017). Most of the existing JD-R studies assess job demands and resources at the individual level. However, schools vary in their organizational climates, interpersonal relationships among colleagues, and expectations on teachers' emotional management. Therefore, based on a recent development in JD-R theory (Bakker and Demerouti, 2014, 2017; Schaufeli and Taris, 2014), the present study first adjusts the JD-R model by including personal resources and personal demands. Then, using multilevel analyses, it examines the relationships between teacher well-being and some job-related factors at individual (i.e., emotion regulation strategies) and school levels (i.e., the emotional job demands of teaching and trust in colleagues), in order to obtain a more comprehensive understanding of the predictors of teacher well-being in school settings.

## Theory and hypotheses

### The JD-R model and its recent development

As a heuristic and flexible framework for considering how job characteristics influence individual well-being and work performance, the JD-R model is popular for its inclusiveness in defining job characteristics and the dual processes of its mechanism (Bakker and Demerouti, 2014). According to Bakker and Demerouti (2007), job demands are the physical, psychological, social, or organizational aspects of the job that require sustained effort or skill and are therefore associated with certain costs. Examples of job demands are work overload, emotional demands, and job insecurity. In contrast, job resources are the physical, psychological, social, or organizational aspects of the job that may “be functional in achieving work goals; reduce job demands and the associated physiological and psychological costs; stimulate personal growth and development” (p. 312). Examples of job resources are performance feedback, job control, and social support. They may be located in the organization at large, interpersonal and social relations, or tasks.

Moreover, the JD-R model contains two fairly independent psychological processes influencing individuals' well-being and performance: a health impairment process and a motivational process (Bakker and Demerouti, 2007). The health impairment process assumes that poorly designed jobs or chronic job demands exhaust individuals' physical and psychological resources and may therefore cause burnout, health problems, and poor work performance. Meanwhile, the motivational process suggests that job resources may serve as both intrinsic and extrinsic motives, because job resources can fulfill basic human needs such as autonomy, competence, and relatedness, or create a supportive work environment that leads to high work engagement and excellent performance (Bakker and Demerouti, 2007, 2014).

Since its development about 15 years ago, the hypotheses of JD-R model have been extensively applied and generally supported in fields such as organizational behavior (Schaufeli and Bakker, 2004; van Emmerik et al., 2009), occupational psychology (Demerouti et al., 2001; Bakker et al., 2005), and human resource management (Bakker et al., 2004; Van De Voorde et al., 2016). In the context of schooling, Hakanen et al. (2006) examined the two parallel processes involved in teachers' burnout and engagement with a sample of Finish teachers, and found that although both processes existed the health impairment process seemed to be more prominent. Simbula's (2010) multilevel analysis of teachers' well-being indicators (engagement, mental health, and job satisfaction) also confirmed the dual processes of the JD-R model and revealed the dynamic psychological processes that determined daily fluctuations in teacher well-being.

As popular and robust as the original JD-R model is, a few recent reviews pointed out ways to extend it (Bakker and Demerouti, 2014, 2017; Schaufeli and Taris, 2014). The most salient development of JD-R theory is the integration of personal characteristics (Bakker and Demerouti, 2007, 2014). Since human behavior results from the interaction between personal and environmental factors, integrating the personal characteristics into the original model may enhance its explanatory power (Xanthopoulou et al., 2007, 2009; Schaufeli and Taris, 2014). Following this line of inquiry, quite a few studies examined the roles of personal resources in the JD-R model (Schaufeli and Taris, 2014). Personal resources are the psychological characteristics or aspects of the self that refer to individuals' ability to control and impact their environment successfully (Xanthopoulou et al., 2007; Bakker and Demerouti, 2014; Schaufeli and Taris, 2014). Similar to job resources, personal resources were also found to play positive roles in stimulating individuals' work engagement and buffering the negative impact of job demands. However, the influencing mechanism of these personal resources within JD-R model varies across studies (Xanthopoulou et al., 2007, 2009; Brenninkmeijer et al., 2010; Schaufeli and Taris, 2014; Huang et al., 2016). For example, Xanthopoulou et al. (2009) found that personal resources directly predicted later work engagement, next to job resources. Huang et al. (2016) revealed that personal resources significantly mediated the relationship between job characteristics and employees' burnout. In contrast, Brenninkmeijer et al. (2010) suggested that personal resources significantly moderated the effects of job characteristics on teachers' emotional exhaustion and work engagement.

As the counterpart of personal resources, “personal vulnerability factors” (Schaufeli and Taris, 2014, p. 57) or “personal demands” (Bakker and Demerouti, 2017, p. 7), however, have not receive enough attentions in this line of inquiry. These personal vulnerability factors may simply be viewed as individuals' *inability* to control and impact their environment successfully (the opposite side of personal resources), or, in Bakker and Demerouti's (2017) terms, as personal demands or “requirements that individuals set for their own performance and behavior that force them to invest effort in their work and are therefore associated with physical or psychological costs” (p. 7). Thus, following the conceptualization of job demands, personal demands could be seen as individuals' characteristics that require *extra* effort or skills and are associated with extra costs. The extra effort or costs could due to individuals' inability/vulnerability (e.g., neuroticism, and pessimism, Schaufeli and Taris, 2014) or due to their higher expectation on their behaviors and performance (e.g., self-demanding, Bakker and Demerouti, 2017; workaholism, Schaufeli and Taris, 2014).

Another unresolved area in JD-R theory is the multilevel issue (Schaufeli and Taris, 2014; Bakker and Demerouti, 2017). Although the method of multilevel analysis has been adopted in a few recent studies (e.g., Xanthopoulou et al., 2009; Simbula, 2010), job demands and resources are usually assessed at the individual level in literature on the JD-R model. However, when we try to examine the impacts of both individual and environmental factors on individual outcomes, it is more appropriate to differentiate the individual-level and organizational-level impacts by using hierarchical linear modeling, especially when participants are nested within different contextual units. For example, schools in Hong Kong vary in cultural traditions. In Hong Kong, each school has its own sponsoring body, which might be a religious group (e.g., Catholic, Christian, Buddhist, Taoist, etc.), a charitable organization (e.g., Po Leung Kuk, Hong Chi Association, etc.) or the Hong Kong government. Therefore, schools in Hong Kong have various expectations on teachers' emotional expressions and different mechanisms for teachers to deal with their emotional problems. Teachers from the same school face with the same demanding and supportive environment. This study thus examines the relationships between teacher well-being and its predictors at the school and individual levels using hierarchical linear modeling. Specifically, at the school level, the emotional job demands of teaching and trust in colleagues are taken as the job demand and resource, respectively; at the individual level, the emotion regulation strategies of reappraisal, and suppression are considered as the personal resource and demand, respectively.

**Figure 1:**
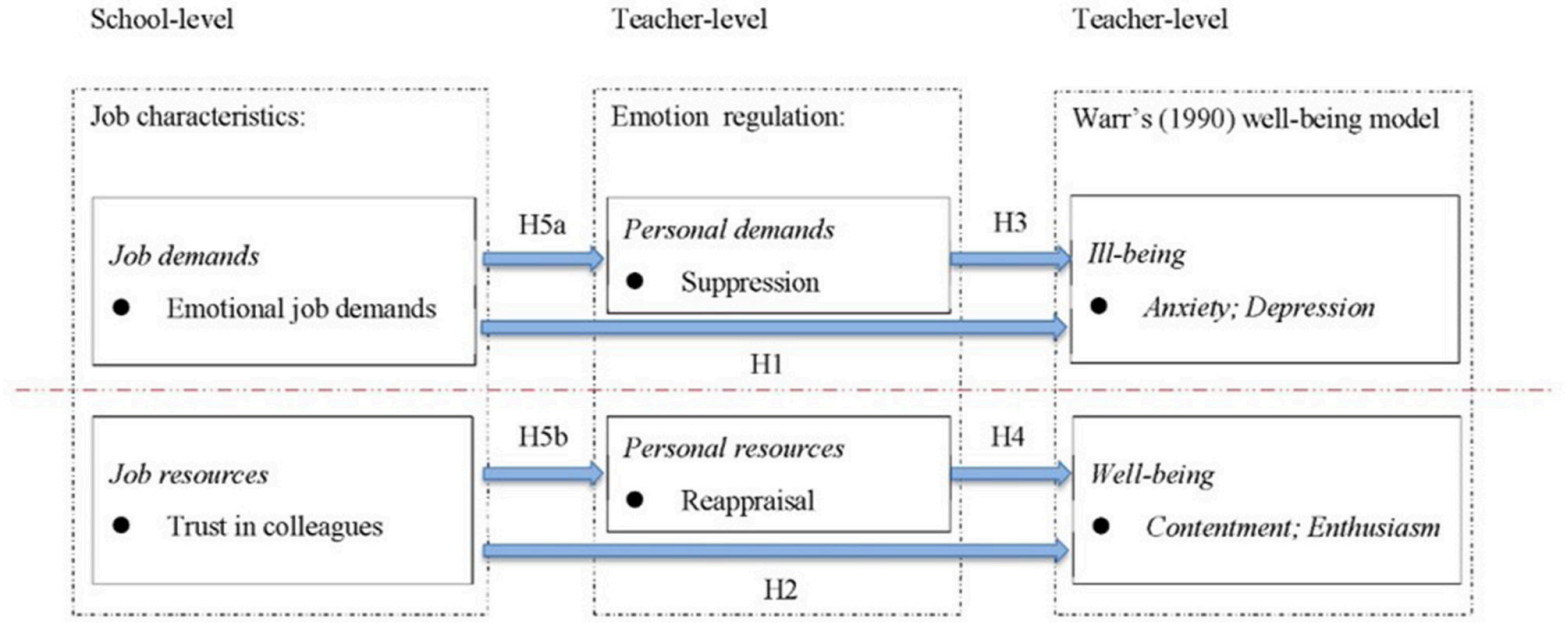
Shows the hypothesized model tested in this study.

Further, although the health impairment and motivational processes in the original JD-R model are assumed to be fairly independent, as Schaufeli and Taris (2014) suggested, they represent “two sides of the same coin” and therefore should be studied jointly (p. 57). However, most studies of the JD-R model have used contrasting and fragmented well-being indicators such as exhaustion/satisfaction and burnout/engagement, and the motivational process has often been examined in isolation (e.g., Xanthopoulou et al., 2007, 2009; Huang et al., 2016). By adopting Warr's (1990) model, the present study is able not only to confirm the positive relationship between demands and burnout (deactivated ill-being, cf. depression) and that between resources and engagement (activated well-being, cf. enthusiasm), but also to explore the impacts of these individual and organizational factors on activated ill-being (anxiety) and deactivated well-being (contentment) following the health impairment and motivational processes of the JD-R model (Schaufeli and Taris, 2014). Specific research hypotheses (see Figure 1) are established in the following sections.

### The emotional job demands of teaching and trust in colleagues as school-level predictors

Emotional job demands are qualitative demands imposed by the frequency, intensity, and variety of interpersonal interactions required by the job (Brotheridge and Lee, 2002). They lead to sustained personal effort and are associated with certain costs (Bakker and Demerouti, 2007). In Yin's (2015) terms, the emotional job demands of teaching relate to the requirement that teachers manage their emotions due to their intense and frequent interactions with colleagues, students, and parents.

According to the health impairment process of the JD-R model, excessive job demands drain individuals' physical and/or psychological energies, and the constant depletion of these energies leads to burnout or worsens health problems (Bakker and Demerouti, 2007, 2014). Emotional job demands are usually considered stressful and detrimental not only because meeting them may deplete resources that people value (Hobfoll, 1989; Brotheridge and Lee, 2002), but also because they may lead to the unpleasant feeling that one's emotions are beyond one's control (Grandey, 2000).

Studies have demonstrated the positive relationships between emotional job demands and unpleasant outcomes such as burnout, job dissatisfaction, and low commitment (Brotheridge and Lee, 2002; Bozionelos and Kiamou, 2008). It has been shown that job demands were positively related to employees' trait anxiety (Richardsen et al., 1992) or work anxiety (Maria et al., 2018). Emotional job demands were also found to be significantly related to employees' depressive symptoms and emotional exhaustion (Huang et al., 2017; Thuynsma and De Beer, 2017; Maria et al., 2018). In the context of schooling, Yin et al. (2016) also reported that the emotional job demands of teaching were positively related to teachers' emotional exhaustion and negatively related to teaching satisfaction. Thus, it is assumed that emotional job demands relate positively to depression and anxiety following the health impairment process.

H1: The emotional job demands of teaching are positively related to depression and anxiety.

Trust has been suggested as the “taken-for-granted” basis of interpersonal interactions and the “foundation of school effectiveness” (Louis, 2007), but little work has been done to investigate the relationship between trust and teacher well-being in schools. According to Mayer et al. (1995), trust is “the willingness of a party to be vulnerable to the actions of another party based on the expectation that the other will perform a particular action important to the trustor, irrespective of the ability to monitor or control that other party” (p. 712). In the context of schooling, teachers' perception of trust in their colleagues depends on the extent to which they believe their colleagues are benevolent, reliable, competent, honest, and open (Tschannen-Moran and Hoy, 2000).

According to the motivational process of the JD-R model, job resources, such as job autonomy, supportive colleagues, and feedback from others, may serve as both extrinsic and intrinsic motives and lead to higher work engagement and better performance (Bakker and Demerouti, 2007, 2014). A trusting relationship between colleagues is the foundation of teacher collaboration (Mayer et al., 1995) and encourages teachers to share their feelings openly and interpret others' behavior in good faith (Dirks and Ferrin, 2001), which lightens individuals' workloads and relieves their emotional strain. Unlike social support, which depicts how colleagues help each other with their work-related questions or to reduce work pressure (e.g., Chen et al., 2009), trust in colleagues plays a more fundamental role in interpersonal relationships because it is the premise of any collaborative activities among people and reflects the emotional bonds among school teachers (Troman, 2000).

Empirical studies have indicated a positive role of trust in colleagues in teachers' work. For instance, it has been reported that trust in colleagues has beneficial effects on teachers' efficacy and commitment (Lee et al., 2011), in addition to their perceptions of job satisfaction and reduced burnout (Van Maele and Van Houtte, 2012). Meanwhile, a low-trust climate in schools may increase teachers' stress and hostile emotions (Troman, 2000). As Casciaro (2014) pointed out, trust is likely to be saturated with affective content because it denotes bonds between individual founded on genuine care and concern for the welfare of partners. In the presence of a trusted party, a person may experience positive affect and be excited and enthusiastic (Jones and George, 1998). Thus, it is reasonable to assume:

H2: Trust in colleagues is positively related to enthusiasm and contentment.

As mentioned before, in this study, teachers' perception of the emotional job demands of teaching and their perception of trust in colleagues are seen as a job demand and a resource, respectively, at the school level.

### Emotion regulation strategies as individual-level predictors

In Huang, Wang and You (2016) words, emotion regulation denotes “the processes by which individuals influence which emotions they have, when they have them, and how they experience and express their emotions” (p. 275). The difference between the two common types of emotion regulation strategies, reappraisal, and suppression, lies in the timing of individual interventions. Reappraisal occurs before the arousal of emotions induced by external stimuli, while suppression comes after the formation of specific emotions (Gross, 2015).

Emotion regulation reflects individuals' ability to cope with stress-provoking situations and control their emotions in the workplace (Buruck et al., 2016; Yin et al., 2016). Further, according to Joseph and Newman (2010), individuals with high ability to regulate emotion often engage in more effective strategy (cognitive reappraisal) while those adopting the strategy of suppression exhibit a low emotion regulation competence. Empirically, Gross and John (2003) reported that reappraisal strategies were associated with better interpersonal functioning and were positively related to well-being, whereas suppression strategies were associated with worse interpersonal functioning and were negatively related to well-being. Wallace et al. (2009) found that suppression consumed more resources and was negatively related to task performance, while reappraisal saved more resources and was positively related to task performance. Buruck et al. (2016) also pointed out that reappraising the emotional stimulus was a proven effective way to deal with stress-provoking situations, whereas suppression of negative emotional behavior was less effective and could have a negative impact on individuals. Thus, in accord with previous definitions of personal resources and personal demands/vulnerability factors (Schaufeli and Taris, 2014; Bakker and Demerouti, 2017), reappraisal should be considered a personal resource, which refers to individuals' ability to efficiently control their emotions and to adapt themselves to environment, whereas suppression should be seen as a personal demand, which reflects individuals' inability to cope with the emotional demanding environment and hence is associated with extra effort and physical or psychological costs (Bakker and Demerouti, 2017).

In school settings, similar patterns in the relationships between emotion regulation strategies and teachers' well-being indicators have also been detected. It has been argued that reappraisal may be more adaptive and effective than suppression in the classroom (Fried, 2011; Jiang et al., 2016; Yin, 2016). Recent empirical studies of teachers' emotion regulation have also consistently shown that suppression plays a detrimental role in influencing teacher well-being, whereas reappraisal has beneficial effects on well-being indicators such as enjoyment and job satisfaction (Kafetsios et al., 2012; Jiang et al., 2016; Yin et al., 2016).

According to the recent development of the JD-R model and the empirical research summarized previously, it is reasonable to assume that reappraisal, as a personal resource, plays a desirable role in influencing teacher well-being and is related to pleasant outcomes, while suppression, as a personal demand, is detrimental to teacher well-being and associated with unpleasant outcomes.

H3: Suppression is positively related to depression and anxiety.
H4: Reappraisal is positively related to enthusiasm and contentment.

## Relationships between school- and individual-level predictors

While the environmental and personal factors together impact human behaviors and outcomes, another important issue concerns with the connections between school- and individual-level predictors. On the one hand, choosing to use reappraisal or suppression is a behavior tendency in individuals and reflects their ability/inability to regulate their own emotion (Gross and John, 2003; Joseph and Newman, 2010). In short term, this tendency or ability is more likely to be changed or fostered by pre-designed interventions than by daily environmental stimuli. On the other hand, the usage of emotional regulation strategies is also a reaction to the supportive or demanding environment. The emotional job demands of teaching are the preconditions that make the ability to regulate emotions (reappraisal) an advantage while the lack of ability (suppression) a deficiency. Trust in colleagues and supportive environment, in contrary, encourage teachers to be themselves and make the inability to control emotions (suppression) less a deficiency.

Empirically, Peng et al. (2010) found that the emotional demands of a job increased employees' use of different coping strategies. Employees adopt strategies with which they are more habitual. However, other researchers found more interesting results. For example, Lo's (2002) study of nursing students empirically demonstrated that the stress resulting from the emotional job demands of nursing correlated with the nurses' avoidance coping and negative self-esteem. Employees were found to use suppression strategies or hide their emotions in unfamiliar surrounding or under stress (Grandey, 2000; Joseph and Newman, 2010). Individuals may feel more comfortable being themselves when safety has been ensured (Edmondson, 1999). Therefore, we may hypothesize that emotional job demands of teaching, as external stressors, challenge teachers' emotion regulation ability, and is positively related to suppression. However, trust in colleagues would engender a more reliable environment, which enables teachers to believe in the benevolence of their colleagues and thus reinterpret the poor behavior of others as only accidental rather than intentional (Yin et al., 2016). Therefore, trust in colleagues should be positively related to reappraisal.

H5: The emotional job demands of teaching are positively related to suppression (H5a) while trust in colleagues is positively related to reappraisal (H5b).

## Methods

### Procedures and participants

Consistent with institutional review board procedures, this study was carried out in accordance with the recommendations of Survey and Behavioral Research Ethics Committee at the Chinese University of Hong Kong with written informed consent from all subjects. All subjects gave written informed consent in accordance with the Declaration of Helsinki. The protocol was approved by the Survey and Behavioral Research Ethics Committee.

The participants in the present study were school teachers, and the survey was conducted from November 2015 to March 2016. Invitation letters with copies of the survey questionnaire were sent to the contact persons of 60 primary and 30 secondary schools in different districts, and these persons then helped with recruiting volunteers. A cover letter was also attached to explain the nature, purpose, and procedure of the survey. The completed survey questionnaires were sealed in envelopes and returned directly to the researchers.

Of the 3,000 copies of questionnaires distributed, 1,764 were returned, putting the return rate at 58.8%. After removing invalid questionnaires, the final sample consisted of 1,656 teachers from 54 schools (38 primary and 16 secondary schools), yielding a useful response rate of 55.2%. The sample comprised 1,115 (67.3%) primary school teachers and 541 (32.7%) secondary school teachers. There were 465 male (28.1%) and 1,167 female (70.5%) teachers in the sample, with another 24 participants (1.4%) who did not report their gender.

### Measures

#### Teacher well-being

Warr's (1990) 12-item Affective Well-being Scale was used to measure teacher well-being in the workplace. In this scale, three items are designed to assess teachers' anxiety, contentment, depression, and enthusiasm. Participants were asked to think of the past few weeks and figure out how much of the time their teaching made them feel each of the following: tense, uneasy, and worried (for anxiety); calm, contented, and relaxed (for contentment); depressed, gloomy, and miserable (for depression); and cheerful, enthusiastic, optimistic (for enthusiasm). The Cronbach's alpha coefficients of anxiety, contentment, depression, and enthusiasm were 0.91, 0.81, 0.89, and 0.88, respectively.

#### The emotional job demands of teaching

Yin's (2015) 4-item Emotional Job Demands of Teaching Scale was used in this study. Sample items included the following: “To perform my teaching well, I have to spend most of my time interacting with others (e.g., students, parents, and colleagues)” and “I have to use my emotions and behavior to create a reassuring climate for my students and their parents.” The Cronbach's alpha coefficient of emotional job demands was 0.68.

#### Trust in colleagues

The 5-item Trust in Colleague Scale suggested by Lee et al. (2011) was used in this survey. Sample items included the following: “Even in difficult situations, teachers in this school can rely on each other” and “Teachers in this school have faith in the integrity of their colleagues.” The Cronbach's alpha coefficient of trust in colleagues was 0.88.

#### Emotion regulation

Gross and John's (2003) 10-item Emotional Regulation Questionnaire (ERQ) was used to assess two emotion regulation strategies. Six items were used to measure reappraisal strategies and four items were used to measure suppression strategies. Sample items included the following: “When I want to feel more positive emotions in teaching I change what I'm thinking about” (for reappraisal) and “I control my emotions in teaching by not expressing them” (for suppression). The Cronbach's alpha coefficients of reappraisal and suppression were 0.79 and 0.73, respectively.

#### Control variables

The control variables at the individual level were gender (male coded as “0,” female coded as “1”), education, position in the school, years of teaching experience (coded from low to high), and teachers' self-monitoring. Self-monitoring generally reflects teachers' basic ability to control their expression and behaviors and is measured using the scale developed by Snyder and Gangestad (1986). The control variables at the school level included school type (primary as “0,” secondary as “1”) and display rule perceptions at school. Display rule perceptions were measured by positive display rule perceptions (PDRP; e.g., “Part of my job is to make my students feel good.”) and negative display rule perceptions (NDRP; “I am expected to suppress my bad moods or negative reactions to students.”) subscales developed by Diefendorff et al. (2005). Individuals with high levels of PDRP think that they should show positive emotions, while those with high levels of NDRP believe that they should suppress negative emotions (Grandey, 2000; Diefendorff and Richard, 2003; Diefendorff et al., 2005).

Except stated otherwise, all items were scored on 5-point Likert scales ranging from “1 = strongly disagree” to “5 = strongly agree.” For those scales originally in English, translation and back-translation procedures were followed to convert them into Chinese.

### Analyses

#### Preliminary analyses

The expectation maximum (EM) algorithm in SPSS 22 was first used to handle the missing data. Then, Cronbach's alpha coefficients were calculated using SPSS 22 to confirm the reliability of the scales. For school-level variables (i.e., emotional job demands of teaching, trust in colleagues, and display rule perceptions), individual scores were aggregated to form the school-level data. Finally, descriptive statistics (*M, SD*) and correlations for the variables in each level were calculated.

#### Hierarchical linear modeling

HLM2 in HLM 6.08 was used to merge individual- and school-level data for the two-level analyses. Individual-level variables were centered using group mean centering, while the school-level variables were centered using grand mean centering in each model (Reyes et al., 2012; Woltman et al., 2012). For each criterion, three models were constructed.

The results of the null models provided the value of between-group variance (τ_00_) and within-group variance (σ^2^), while the results of the individual-level-only models and individual- and school-level models provided the value and significance of each parameter (γ_*kl*_/μ_*kl*_) and individual-level residual variance (σ^2^*-adjusted*), school-level variance in intercepts (τ_00_*-adjusted*), and school-level variance in slopes (τ_*kk*_, k = 1,2,3,4,5,6). The between-group variance (τ_00_) was then divided by the total variance (between-group variance + within-group variance, τ_00_ + σ^2^) in teacher well-being to obtain the value of the intra-class correlation (ICC), which represented the percentage of school-level variance in teacher well-being. Effect sizes for each parameter were calculated using the formula $\delta \;=\;\frac{\gamma }{\sqrt{\tau_{00}+{\;\sigma }^{2}}}$ (Reyes et al., 2012). Explained variances (R^2^) were used to identify the proportion of the between- or within-group variance in teacher well-being explained by the predictors (Woltman et al., 2012). Specifically, explained within- and between-group variances were calculated using the formulas ${{\text{R}}^{2}}_{\text{level }1}=\frac{{{\;\sigma }^{2}}_{\text{previous model}}-{{\;\sigma }^{2}}_{\text{current model}}}{{{\;\sigma }^{2}}_{\text{previous model}}}$ and ${{\text{R}}^{2}}_{\text{level }2}=\frac{\tau_{00\text{ previous model}}-\tau_{00\text{ current model}}}{\tau_{00\text{ previous model}}}.$

## Results

### Descriptive statistics and correlations

Before further data analyses, the distributive normality of the Level 1 and Level 2 variables was examined by various methods including Skewness, Kurtosis, P-P, and Q-Q plots. The results supported the normality of distribution for all variables.

Table 1 shows the means and standard deviations of the variables and the correlations between them. According to the results of the Level 2 variables, the teachers in secondary schools had lower emotional job demands and display rule perceptions than those in primary schools. Emotional job demands at school were significantly associated with positive and negative display rule perceptions and were not associated with trust in colleagues. Meanwhile, trust in colleagues was positively related to positive display rule perceptions but not to negative display rule perceptions.

**Table 1:** Mean, standard deviation, and correlations for all variables in two-level model.

	***M***	***SD***	**1**	**2**	**3**	**4**	**5**	**6**	**7**	**8**	**9**	**10**	**11**
**LEVEL 2: SCHOOLS (*****N*** = **54)**													
1.EJD	3.91	0.15	(0.68)										
2.Trust	3.58	0.26	−0.01	(0.88)									
3.PDRP	3.83	0.13	0.48^**^	0.46^**^	(0.69)								
4.NDRP	3.41	0.20	0.52^**^	−0.08	0.60^**^	(0.72)							
5.School type	0.33	0.47	−0.35^*^	−0.25	−0.36^**^	−0.39^**^	–						
**LEVEL 1: TEACHERS (*****N*****= 1656)**													
1.Anxiety	2.98	0.90	(0.91)										
2.Depression	2.59	0.95	0.76^**^	(0.81)									
3.Contentment	2.89	0.75	−0.54^**^	−0.55^**^	(0.89)								
4.Enthusiasm	3.06	0.78	−0.43^**^	−0.49^**^	0.75^**^	(0.88)							
5.Reappraisal	3.69	0.46	0.03	0.01	0.08^**^	0.12^**^	(0.79)						
6.Suppression	3.04	0.67	0.25^**^	0.27^**^	−0.18^**^	−0.16^**^	0.30^**^	(0.73)					
7.Gender	0.72	0.45	0.08^**^	0.02	−0.11^**^	−0.08^**^	0.07^**^	−0.09^**^	–				
8.Education	2.38	0.57	0.01	0.02	−0.03	−0.04	0.01	−0.01	0.01	–			
9.Experience	2.85	1.10	−0.11^**^	−0.09^**^	0.12^**^	0.04	0.01	−0.01	−0.07^**^	−0.01	–		
10.Position	1.42	0.64	−0.03	−0.05^*^	0.07^**^	0.04	0.01	−0.02	−0.11^**^	0.11^**^	0.40^**^	–	
11.SelfM	2.99	0.56	0.17^**^	0.16^**^	−0.03	0.03	0.14^**^	0.27^**^	−0.13^**^	0.02	−0.15^**^	−0.01	(0.77)

* p < 0.05;

** *p < 0.01;Cronbach's alpha coefficients in parentheses along the diagonal; EJD, emotional job demands of teaching; Trust, trust in colleagues; PDRP, positive display rule perceptions; NDRP, negative display rule perceptions; SelfM, self-monitoring*.

As for the Level 1 variables, reappraisal was not significantly related to anxiety or depression, but was positively related to enthusiasm and contentment; suppression was positively related to anxiety and depression and negatively related to enthusiasm and contentment. Self-monitoring was positively related to anxiety and depression, but not significantly associated with enthusiasm or contentment. The correlation between self-monitoring and suppression was stronger than that between self-monitoring and reappraisal.

### HLM analyses

Four null models without predicting variables were constructed for anxiety, depression, contentment, and enthusiasm, the ICCs of which were 0.07 (τ_00_ = *0.06*, σ^2^ = 0.75), 0.06 (τ_00_ = *0.05*, σ^2^ = 0.85), 05 (τ_00_ = *0.03*, σ^2^ = 0.54), and 0.04 (τ_00_ = *0.02*, σ^2^ = 0.58), respectively. Similarly, the ICCs for reappraisal and suppression were 0.01 (τ_00_ = *0.002*, σ^2^ = 0.21) and 0.01 (τ_00_ = *0.003*, σ^2^ = 0.44). Although Cohen (1988) suggested that HLM should be used when the value of an ICC is higher than 0.059, there is no consensus on the cut-off value for the ICC, and some researchers believe that theoretical guidance is more important for the decision to use multilevel modeling (Luke, 2004; Woltman et al., 2012). Thus, hierarchical linear modeling was used for the subsequent analyses.

Results for ill-being (anxiety and depression) and for well-being (contentment and enthusiasm) are presented in Tables 2, 3, respectively. Results for reappraisal and suppression are shown in Table 4. The fixed effect is reported first: the first column for each model reports the value and significance of each parameter (γ), and the second column for each model reports the effect size (δ) of each parameter and the standard error (*SE*, in parentheses). Then, the random effect and explained variance are also reported. The interpretation of δ is similar to that of Cohen's (1988) *d*: 0.20 is small, 0.50 is moderate, and 0.80 is large.

**Table 2:** Multilevel estimates for models predicting anxiety and depression.

**Independent variable**	**Dependent variables**							
	**Anxiety(ICC** = **0.07)**				**Depression (ICC** = **0.06)**			
	**γ**	**δ(*SE*)**	**γ**	**δ(*SE*)**	**γ**	**δ(*SE*)**	**γ**	**δ(*SE*)**
**FIXED EFFECTS**								
Intercept	3.00^***^	3.33 (0.04)	3.00^***^	3.33 (0.03)	2.61^***^	2.75 (0.04)	2.61^***^	2.75 (0.03)
**Level 1**								
**level 1 covariates**								
Gender	0.19^**^	0.21 (0.06)	0.20^**^	0.22 (0.06)	0.11	0.12 (0.07)	0.11	0.12 (0.07)
Education	0.03	0.03 (0.04)	0.03	0.03 (0.04)	−0.01	−0.01 (0.04)	−0.01	−0.01 (0.04)
Experience	−0.06^*^	−0.07 (0.02)	−0.06^*^	−0.07 (0.02)	−0.03	−0.03 (0.03)	−0.03	−0.03 (0.03)
Position	0.01	0.01 (0.04)	0.01	0.01 (0.04)	−0.06	−0.06 (0.05)	−0.06	−0.06 (0.05)
selfM	0.13^**^	0.14 (0.04)	0.13^**^	0.14 (0.04)	0.13^**^	0.14 (0.04)	0.13^**^	0.14 (0.04)
**Level 1 predictor**								
Su	0.29^***^	0.32 (0.03)	0.29^***^	0.32 (0.04)	0.33^***^	0.35 (0.03)	0.33^***^	0.35 (0.03)
*Level 2*								
*level 2 covariates*								
School type			−0.14^*^	−0.16 (0.07)			0.01	0.01 (0.07)
PDRP			−0.10	−0.11 (0.21)			−0.35	−0.37 (0.22)
NDRP			0.36^*^	0.40 (0.17)			0.31	0.33 (0.21)
*Level 2 predictor*							
EJD			0.95^***^	1.06 (0.17)			1.13^***^	1.19 (0.22)
**RANDOM EFFECT**								
*Level 1(σ2)*		0.66		0.66		0.74		0.74
*Level 2(τ00)*		0.06		0.03		0.06		0.03
**EXPLAINED VARIANCE**								
*Level 1*		12.71%				12.73%	
*Level 2*				54.99%				45.04%

* p < 0.05;

** p < 0.01;

*** *p < 0.001; EJD, emotional job demands of teaching; Trust, trust in colleagues; PDRP, positive display rule perceptions; NDRP, negative display rule perceptions; SelfM, self-monitoring*.

**Table 3:** Multilevel estimates for models predicting contentment and enthusiasm.

**Independent variable**	**Dependent variables**							
	**Contentment (ICC** = **0.05)**				**Enthusiasm (ICC** = **0.04)**			
	**γ**	**δ(*SE*)**	**γ**	**δ(*SE*)**	**γ**	**δ(*SE*)**	**γ**	**δ(*SE*)**
**FIXED EFFECTS**								
Intercept	2.87^***^	3.82 (0.03)	2.87^***^	3.82 (0.03)	3.05^***^	3.92 (0.03)	3.05^***^	3.92 (0.03)
**Level 1**								
**Level 1 covariates**								
Gender	−0.17^***^	−0.23 (0.04)	−0.17^***^	−0.23 (0.04)	−0.15^**^	−0.19 (0.04)	−0.15^**^	−0.19 (0.04)
Education	−0.02	−0.03 (0.03)	−0.02	−0.03 (0.03)	−0.04	−0.05 (0.04)	−0.04	−0.05 (0.04)
Experience	0.05^*^	0.07 (0.02)	0.05^*^	0.07 (0.02)	0.01	0.01 (0.02)	0.01	0.01 (0.02)
Position	0.05	0.07 (0.03)	0.06^*^	0.08 (0.03)	0.05	0.06 (0.03)	0.05	0.06 (0.03)
selfM	−0.04	−0.05 (0.04)	−0.04	−0.05 (0.04)	0.04	0.05 (0.04)	0.03	0.04 (0.04)
**Level 1 predictor**								
Re	0.15^***^	0.20 (0.04)	0.16^***^	0.21 (0.04)	0.22^***^	0.28 (0.05)	0.22^***^	0.28 (0.05)
**Level 2**								
**Level 2 covariates**								
School type			0.03	0.04 (0.07)			−0.05	−0.06 (0.06)
PDRP			−0.71^*^	−0.95 (0.35)			−0.52	−0.67 (0.39)
NDRP			−0.01	−0.01 (0.20)			−0.02	−0.03 (0.21)
**Level 2 predictor**								
Trust			0.42^*^	0.56 (0.14)			0.47^**^	0.60 (0.15)
**RANDOM EFFECT**								
*Level 1(σ2)*		0.49		0.49		0.52		0.52
*Level 2(τ00)*		0.03		0.02		0.03		0.02
**EXPLAINED VARIANCE**								
*Level 1*		9.01%					10.32%
*Level 2*				27.58%				23.17%

* p < 0.05;

** p < 0.01;

*** *p < 0.001; EJD, emotional job demands of teaching; Trust, trust in colleagues; PDRP, positive display rule perceptions; NDRP, negative display rule perceptions; SelfM, self-monitoring*.

**Table 4:** Multilevel estimates for models predicting reappraisal and suppression.

**Independent variable**	**Dependent variables**							
	**Reappraisal (ICC** = **0.01)**				**Suppression (ICC** = **0.01)**			
	**μ**	**δ(*SE*)**	**μ**	**δ(*SE*)**	**μ**	**δ(*SE*)**	**μ**	**δ(*SE*)**
**FIXED EFFECTS**								
Intercept	3.70^***^	8.08 (0.01)	3.70^***^	8.08 (0.01)	3.05^***^	4.57 (0.02)	3.05^***^	4.57 (0.02)
**Level 1**								
**Level 1 covariates**								
Gender	0.08^**^	0.17 (0.03)	0.09^**^	0.20 (0.03)	−0.10^*^	−0.15 (0.04)	−0.10^*^	−0.15 (0.04)
Education	0.01	0.02 (0.02)	0.01	0.02 (0.02)	−0.01	−0.01 (0.03)	−0.01	−0.01 (0.03)
Experience	0.01	0.02 (0.01)	0.01	0.02 (0.01)	0.04^**^	0.06 (0.01)	0.04^**^	0.06 (0.01)
Position	0.01	0.02 (0.02)	0.01	0.02 (0.02)	−0.07^**^	−0.10 (0.02)	−0.07^**^	−0.10 (0.03)
selfM	0.11^***^	0.24 (0.02)	0.11^***^	0.24 (0.02)	0.32^***^	0.48 (0.03)	0.32^***^	0.48 (0.03)
**Level 2**								
**Level 2 covariates**								
School type			−0.03	−0.07 (0.03)			0.02	0.03 (0.03)
PDRP			0.35^*^	0.76 (0.14)			−0.21	−0.31 (0.16)
NDRP			−0.11	−0.24 (0.09)			0.28^**^	0.42 (0.09)
**Level 2 predictor**								
EJD							0.31^*^	0.46 (0.12)
Trust			0.01	0.02 (0.07)			
**RANDOM EFFECT**								
*Level 1(σ2)*		0.19		0.19		0.39		0.39
*Level 2(τ00)*		0.003		0.002		0.005		0.003
**EXPLAINED VARIANCE**								
*Level 1*		8.51%				10.60%	
*Level 2*				33.23%				52.81%

* p < 0.05;

** p < 0.01;

*** *p < 0.001; EJD, emotional job demands of teaching; Trust, trust in colleagues; PDRP, positive display rule perceptions; NDRP, negative display rule perceptions; SelfM, self-monitoring*.

As shown in Tables 2–4, after controlling for individual- and school-level covariates, the hypothesized effects of both individual- and school-level predictors on outcomes were supported, except for the effect of trust in colleagues on reappraisal.

When considering the impacts of individual level variables on teachers well-being and ill-being, it was found that reappraisal was positively related to contentment (δ = *0.20, p*<*0.001*) and enthusiasm (δ = *0.28, p*<*0.001*) whereas suppression was positively related to anxiety (δ = *0.32, p*<*0.001*) and depression (δ = *0.35, p*<*0.001*). These results also indicated that individual-level variables explained 9.01~12.73% of the within-group variance. H3 and H4 were supported.

After adding school-level variables, it was found that emotional job demands of teaching were positively related to anxiety (δ = *1.06, p*<*0.001*) and depression (δ = *1.19, p*<*0.001*) whereas trust in colleagues was positively related to contentment (δ = *0.56, p*<*0.01*) and enthusiasm (δ = *0.60, p*<*0.05*). The school-level variables explained 23.17~54.99% of the between-group variance. H1 and H2 were supported.

As for the impacts of school level predictors on reappraisal and suppression, it was found that emotional job demands of teaching were positively related to suppression (δ = *1.06, p*<*0.001*) while the relationship between trust in colleagues and reappraisal was non-significant. H5a was supported but H5b was not.

## Discussion

By integrating personal demands and resources into the JD-R model, this study examined the relationships between emotional job demands of teaching, expressive suppression, and teacher ill-being and those between trust in colleagues, cognitive reappraisal, and teacher well-being. The results of the multilevel analyses fully supported all of the hypotheses except H_5b_, which was not supported due to the non-significant relationship between school-level trust in colleagues and teachers' reappraisal. In general, the results indicated that, when faced with high emotional job demands, teachers tended to use suppression strategies and feel more anxiety, and depression following the health impairment process. Meanwhile, it was also found that, following the motivational process, trust in colleagues and the adoption of reappraisal strategy were positively related to contentment and enthusiasm. These findings shed light on the JD-R model, teachers' emotion regulation, and well/ill-being in schools.

### Theoretical implications

First, the JD-R model assumes that job demands are positively related to burnout following the health impairment process while job resources are positively associated with engagement following the motivational process (Demerouti et al., 2001; Bakker and Demerouti, 2007). By adopted a two-axis comprehensive model of affective well-being (Warr, 1990), this present study is able to confirm the positive relationships between emotional job demands and both activated and deactivated states of ill-being (anxiety and depression) and those between trust in colleagues and both states of well-being (enthusiasm and contentment). The results are consistent with the hypotheses of the JD-R model and expand our current knowledge on the detrimental roles of job demands and the beneficial one of job resources.

Second, although the multilevel issue of the JD-R model has repeatedly been suggested as a direction for future research (Schaufeli and Taris, 2014; Bakker and Demerouti, 2017), job demands and resources are usually investigated at the individual level. By integrating personal factors into the JD-R model, the multilevel analyses in the present study showed that school- and individual-level predictors played important roles in explaining the variance in teacher well-being. Specifically, the individual-level predictors (reappraisal and suppression) explained 9.01~12.73% of the within-group variance, and the school-level predictors (the emotional job demands of teaching and trust in colleagues) explained 23.17~54.99% of the between-group variance. These results highlighted the significance of multilevel analysis in research using the JD-R model. Moreover, at the school level, the health impairment process was found to be much more prominent than the motivational process: the emotional job demands of teaching explained 54.99 and 45.04% of the between-group variance of anxiety and depression, respectively. In line with Hakanen et al. (2006), these results indicated that teachers may be more sensitive to working conditions that translate into losses for them.

Third, the integration of personal resources has been identified as the most salient development of the JD-R model (Bakker and Demerouti, 2007, 2014; Schaufeli and Taris, 2014), and a number of studies in the past decade have explored the role of personal resources in applied research (e.g., Xanthopoulou et al., 2007, 2009; Brenninkmeijer et al., 2010; Huang et al., 2016). However, the role of personal demands has rarely been examined. In this study, we conceptualize personal demands from an efforts-requirement view and synthesize two similar but different concepts of individuals' inability/vulnerability (Schaufeli and Taris, 2014) and personal high expectations (self-demanding, Bakker and Demerouti, 2017). The study findings confirmed the positive relationship between reappraisal, as a personal resource, and teacher well-being and that between suppression, as a personal demand, and teacher ill-being. These findings lend credence to the practicability of integrating personal resources and demands into the origin JD-R model (Schaufeli and Taris, 2014; Bakker and Demerouti, 2017).

Four, in contrast to Gross's (1998, 2015) stress on the timing of individual interventions, the findings of the present study highlight the differences between reappraisal and suppression from the perspective of personal demands and resources. According to stress and coping theory (Lazarus, 1993), both reappraisal and suppression can be considered strategies for coping with emotionally stressful conditions. However, the results of the present study reveal the distinctions between the two coping strategies. Specifically, reappraisal should be taken as a personal resource that is associated with resiliency and reflects individual teachers' ability to control their environment (Schaufeli and Taris, 2014). In contrast, suppression should be considered as a personal demand. The use of suppression reflects the lack of internal regulatory ability of individual teachers, and hence requires extra effort of teachers in their teaching (Bakker and Demerouti, 2017). In our opinion, to conceptualize reappraisal as a personal resource and suppression a demand may help to explain why reappraisal appears more adaptive and effective than suppression as a way for teachers to manage their emotions in the classroom (Fried, 2011; Jiang et al., 2016; Yin, 2016). However, when examining the impacts of school- level predictors on personal resources and demands, only the positive relationship between emotional job demands and suppression was found to be significant. These findings again highlight the relative prominent role of the health impairment process: the emotional job demands of teaching may push teachers to use maladaptive strategy of suppression but trust in colleagues is not able to improve individuals coping skills.

Last but not least, considering the adoption of 5-point Likert scales, our results showed that teachers' self-reported use of emotion regulation strategies were quite often, especially for the reappraisal strategies (*M* = 3.69, *SD* = 0.46), indicating that emotion regulation is prevalent in Hong Kong teachers' work. This may be related to the cultural contexts in Hong Kong. In the case of the Chinese who live in collectivist cultures emphasizing interdependence of self and social harmony, they may feel and express more other-focused emotions (Yin and Lee, 2012). Anolli et al's. (2008) cross-cultural comparison found that the vocal expression of emotions of the Chinese is characterized by a more restrained style than that of their Italian counterparts. This is because the Chinese culture emphasizes relational harmony and concerns about the impact that emotional practices may have on others. Therefore, appropriate use of emotion regulation strategies is encouraged by the Chinese culture. These cultural issues are worthy of more research in future.

### Limitations and directions for future research

There are two limitations to the present study. First, the survey was carried out at a single point in time. This one-off cross-sectional design made it impossible to determine causal relationships between the constructs of interest in the study. Reciprocal relationships between them may also exist (Xanthopoulou et al., 2009; Schaufeli and Taris, 2014). For example, teachers' well-being status may influence their perceptions of job demands or trust relationships in the workplace and may also affect their adoption of emotion regulation strategies. This limitation indicates a need for longitudinal design in future work.

Second, although this study examined the direct effects of these school- and individual-level predictors of teacher well-/ill-being and the relationships between school- and individual level predictors, some researchers have pointed out that interactions between school- and individual-level demands and resources may exist, and that personal factors may mediate or moderate the relationships between job characteristics and well-being (Xanthopoulou et al., 2007; Schaufeli and Taris, 2014; Bakker and Demerouti, 2017). Therefore, it is recommended that future studies explore the interactions between the school- and individual-level predictors of teacher well-being.

Third, due to the focus of this study, we only examined the moderation effects of two school-level factors on the relationships between emotion regulation and teacher well-being. We also found that years of teaching experience is significantly, though weakly, correlated with three of the four teacher well-being indicators. There exist other potential moderators, such as aging and work experience (Avanzi et al., 2012). Future research is expected to explore other moderators of the relationships between emotion regulation and teacher well-being in the workplace, and the potential moderation effects of aging and work experience.

### Practical implications

Teaching is an emotional endeavor that significantly influences teachers' stress levels and well-being (Sutton, 2004; Chang, 2009, 2013; Yin, 2016). The results of this study not only support the applicability of the JD-R model to school settings, but also suggest some ways to improve teacher well-being in practice. According to Hakanen et al. (2006), job demands appear to be “givens” in the work environment that are essentially inherent to the workplace, while job resources, and personal factors are “alterables” that can change in the short term. This means that improvements in teacher well-being should start from consideration of both the “givens” and “alterables” in schools.

On the one hand, the findings of the present study show that at the school level, the health impairment process appears to be more prominent and the emotional job demands of teaching have significant negative effects on teacher well-being. However, teacher education programs usually focus on knowledge, thinking and teaching techniques, ignoring the emotional demands of teaching (Fried, 2011; Yin and Lee, 2012). Although it is difficult to change teachers' perception of emotional job demands, teacher education programs can make these emotional demands visible to in-service teachers and teacher candidates. A comprehensive understanding of the demands of teaching may make teachers more aware of the influence of teaching on their well-being and thus help them to take effective action.

On the other hand, following Bakker and Demerouti's (2014, 2017) suggestion, teacher well-being can be improved by proactive job crafting interventions aimed at changing the “alterables” in schools and individual teachers. Based on the present study's findings, schools are advised to reinforce an atmosphere of trust by increasing mutual understanding between colleagues and providing opportunities for interpersonal interactions between staff. Meanwhile, considering the significant influence of emotion regulation on teacher well-being and the different roles of various strategies, teachers should be aware of the effects of various emotion regulation strategies and make use of antecedent-focused strategies (i.e., reappraisal) in their work. In this respect, Sutton and Harper (2009) provided good examples of teachers' emotion regulation at five stages, from emotional stimuli to emotional expression; Yin (2016) also summarized seven specific strategies for teachers to regulate their emotions in schools. These studies could serve as a foundation for designing job crafting interventions for teachers.

The purpose for enlarging the repertoire of teachers' knowledge about emotion regulation strategies goes beyond the protection of their personal well-being. Schools and teachers strive for improving the effectiveness of classroom teaching. In fact, teachers' affective well-being is “a necessary condition for teachers' sense of effectiveness” (Day and Gu, 2009, p. 15). As revealed in several previous studies, teachers believe that the emotion regulation strategies, if used appropriately, can be helpful to motivate student learning, facilitate classroom management, and promote their teaching effectiveness (Sutton, 2004; Sutton and Harper, 2009; Yin, 2016). Hence, for sake of effective classroom teaching and personal well-being, teachers should improve their ability of emotion regulation in the workplace.

## Author contributions

HY designed the research, and finished the final version of the manuscript. SH analyzed the data and wrote the first version of the manuscript. LL collected the data and helped with the data analysis.

### Conflict of interest statement

The authors declare that the research was conducted in the absence of any commercial or financial relationships that could be construed as a potential conflict of interest.
